# MCM8 promotes lung cancer progression through upregulating DNAJC10

**DOI:** 10.1111/jcmm.18488

**Published:** 2024-06-21

**Authors:** Lei Cao, Hongsheng Liu, Zhijun Han, Cheng Huang, Chao Guo, Luo Zhao, Chao Gao, Yuan Xu, Guige Wang, Zhe Feng, Shanqing Li

**Affiliations:** ^1^ Department of Thoracic Surgery Peking Union Medical College Hospital, Peking Union Medical College and Chinese Academy of Medical Sciences Beijing China; ^2^ Department of Thoracic Surgery Beijing Sixth Hospital Beijing China

**Keywords:** cell proliferation, DNAJC10, lung cancer, MCM8, migration

## Abstract

MCM8 is a helicase, which participates in DNA replication and tumorigenesis and is upregulated in many human cancers, including lung cancer (LC); however, the function of MCM8 in LC tumour progression is unclear. In this study, we found that MCM8 was expressed at high levels in LC cells and tissues. Further, MCM8 upregulation was associated with advanced tumour grade and lymph node metastasis, and indicated poor prognosis. Silencing of MCM8 suppressed cell growth and migration in vitro and in vivo, while ectopic MCM8 expression promoted cell cycle progression, as well as cell migration, proliferation, and apoptosis. Mechanistically, DNAJC10 was identified as a downstream target of MCM8, using gene array and CO‐IP assays. DNAJC10 overexpression combatted the inhibitory activity of MCM8 knockdown on LC progression, while silencing DNAJC10 alleviated the oncogenic function of MCM8 overexpression. MCM8 expression was positively correlated with that of DNAJC10 in LC samples from The Cancer Genome Atlas database, and DNAJC10 upregulation was also associated with poor overall survival of patients with LC. This study indicated that MCM8/DNAJC10 axis plays an important role in in LC development, and maybe as a new potential therapeutic target or a diagnostic biomarker for treating patients with LC.

## 1 INTRODUCTION

Lung cancer (LC) is among the most commonly diagnosed cancers and a principal cause of cancer‐related mortality globally, with annual new cases and deaths estimated at 2 million and 1.76 million, respectively.^1^, ^2^, ^3^ One reason for the high death rate of patients with LC is that it is often diagnosed at a late stage^4^; the survival rate of regional stage patients is only 27%, and that of distant stage patients is as low as 4%.^5^, ^6^ LC is classified into adenocarcinoma, squamous carcinoma, small cell LC, and non‐small cell LC (NSCLC),^7^ of which NSCLC is the most common form, accounting for approximately 80%–85% of cases.^8^ The mechanisms underlying the various types of LC are entirely different, and they consequently require different treatment strategies and have varying prognosis. Thus, identification of novel biomarkers for early diagnosis of different LCs or to predict prognosis is crucial and urgently required to decrease the death rate of patients with LC.

Minichromosome maintenance (MCM) molecules have significant roles in DNA replication and the cell cycle; there are 10 MCM family members: MCM1–10.^9^ MCM2–7 comprise a DNA helicase subfamily involved in DNA synthesis,^10^ MCM9 and MCM8 are crucial to effective homologous recombination,^11^ and MCM1 and MCM10 are independent members of the family that also function in DNA synthesis.^10^ Many MCM molecules have been reported as diagnostic and prognostic biomarkers in various human cancers, including gastric cancer, hepatocellular carcinoma, cervical cancer, and oesophageal squamous cell carcinoma.^12^, ^13^, ^14^, ^15^, ^16^

A number of studies have shown that minichromosome maintenance 8 homologous recombination repair factor (MCM8) can promote malignant tumour progression.^17^, ^18^, ^19^ MCM8 promotes cholangiocarcinoma progression through regulating PI3K/Akt and MAPK9 signalling.^17^ Further, MCM8 knockdown induces G0/G1 cell cycle arrest and apoptosis, as well as suppressing self‐renewal and proliferation of glioma stem cells (GSCs), while MCM8 knockdown improves GSC sensitivity to radiation and Temozolomide treatments.^18^ MCM8 is expressed at high levels in osteosarcoma tissues, and associated with more advanced tumour grade and pathological stage; silencing of MCM8 through regulating CTGF can suppress osteosarcoma progression.^19^

In LC, MCM8 shows high expression in lung adenocarcinoma (LUAD) tumour tissues from The Cancer Genome Atlas (TCGA), and upregulation of MCM8 is associated with poor overall survival (OS) of patients with LUAD^20^, ^21^; however, the function of MCM8 in LC development is unknown, and was the focus of this study.

## 2 MATERIALS AND METHODS

### 2.1 Samples from The Cancer Genome Atlas (TCGA) and clinical patients

MCM8 expression was analysed in LC samples from TCGA, including data from 526 LUAD tumour and 59 normal lung tissues, and a further 501 LUSC samples with 49 corresponding normal tissues. Data from 476 patients with LUAD, including comprehensive prognostic information, were clustered into high or low expression groups, based on median *MCM8* expression values, or according to *DNAJC10* expressions. OS analysis between high or low groups were conducted using the Kaplan–Meier method with log‐rank test.

A total of 294 NSCLC and 28 normal tissue samples were acquired from Peking Union Medical College Hospital between 2020 and 2021. MCM8 expression was detected using immunohistochemistry (IHC) staining and the relationships between MCM8 expression, tumour stage, and lymph node metastasis assessed. The Ethics Committee of Peking Union Medical College Hospital approved this study, which was conducted according to the Declaration of Helsinki. Written informed consent was obtained from all patients.

### 2.2 Cell culture and infection

Human non‐small cell lung cancer (NSCLC) cell lines (H1299, H1975, and A549) and normal lung epithelial cells (Beas‐2B) were purchased from American Type Culture Collection. Cells were cultured in RPMI‐1640 medium containing 10% fetal bovine serum (FBS) in a 37°C humidified incubator with 5% CO_2_.

Lentiviral mediated knockdown (Vector No, GV115) was used to silence the expression of MCM8 in H1299 and A549 cells. The sequencing of short hairpin RNA molecules (shRNAs) targeting *MCM8* and scrambled shRNA were as follow: shMCM8, 5′‐UCGUAUGAAUAGUCAAGAU‐3′; scrambled shRNA, shCtrl, 5′‐UUCUCCGAACGUGUCACGU‐3′. For the MCM8 overexpression, lentiviral vectors (Vector No, GV287) were employed to overexpress MCM8 (NC_000020.11) in H1975 cells. All lentivirus were purchased from Genechem (Shanghai, China). Cells were seeded in six‐well plate and further incubated for 24 h to reach 70% confluent. Then, cells were infected by above lentivirus with a multiplicity of infection of 30. After 72 h infection, replacing the media, the infected cells were performed the subsequent experiment.

### 2.3 Immunohistochemical (IHC) staining

IHC experiments were conducted using formaldehyde‐fixed, paraffin‐embedded tissue sections. Sections were deparaffinized in xylene, rehydrated in ethanol, and subjected to microwave pretreatment for antigen retrieval in 5 mM Tris‐HCl for 10 min. H_2_O_2_ (3%) was used to quench endogenous peroxidase activity, and an Endogenous Biotin‐Blocking Kit (Thermo Fisher Scientific, USA) was employed to block non‐specific binding sites. Next, slides were incubated with anti‐MCM8 monoclonal primary antibody (1:250; Thermo Fisher Scientific, USA; PA5‐41325), anti‐DNAJC10 (1:250, PA5‐106352; Thermo Fisher) and anti‐Ki67 (1:250, ab16667, Abcam) at 4°C for 4 h, followed by HRP‐conjugated secondary antibody (1:400; Abcam; cat. no. ab6721) at 25°C for 1 h. Samples were visualized using 3,3′‐diaminobenzidine and counterstained with haematoxylin at 25°C for 10 min. An Olympus BX50 microscope (Japan) was used to take representative photographs.

Samples were evaluated by light microscopy using a standard semi‐quantitative immunoreactivity score, as follows: positive staining percentage was scored as: 1 < 25%, 2 = 25%–50%, 3 = 50%–75%, and 4 ≥ 75%; staining intensity was scored as: 0 = negative, 1 = weak, 2 = moderate, and 3 = strong. Positive staining percentage and staining intensity scores were then multiplied to obtained an immunoreactivity score of 0–12, which was used to define low (0–2 scores) and high (2–12 scores) MCM8 expression levels.

### 2.4 Western blot

Total protein samples were extracted from LC cell lines using RIPA lysis buffer (Beyotime), then quantified with a BCA Protein Assay Kit (Pierce). Protein aliquots (20 μg) were separated by 10% SDS‐PAGE, followed by transfer onto PVDF membranes. Membranes were blocked with 5% skimmed milk at 4°C for 1 h. Primary antibodies (dilution, 1:1000) were incubated with membranes at 4°C overnight, as follows: MCM8 (ab191914; Abcam), N‐cadherin (ab76057; Abcam), E‐cadherin (ab231303; Abcam), Vimentin (ab11256; Abcam), anti‐DNAJC10 (PA5‐106352; Thermo Fisher), and GAPDH (an263962; Abcam). Corresponding secondary antibody (1:3000; ab6721; Abcam) was then added, followed by incubation at 25°C for 2 h. The Amersham ECL + TM Western Blot system (Cytiva) was used to visualize protein bands, and signal intensities determined with ImageJ software (version 1.8.0.112, National Institutes of Health).

### 2.5 Reverse transcription‐quantitative PCR (RT‐qPCR)

TRIzol reagent (invitrogen) was used for RNA extraction from transfected cells, and RNA samples were reverse transcribed into cDNA with an M‐MLV Reverse Transcriptase kit (Promega). Then, qPCR was conducted using SYBR‐Green PCR Master Mix (Thermo Fisher Scientific) on a VII7 Sequence Detection system (ABI, USA). The 2^−∆∆Cq^ method was employed to calculate relative mRNA *MCM8* expression.^22^ Primers were *MCM8* forward, 5′‐AATGGAGAGTATAGAGGCAGAGG‐3′ and reverse, 5′‐CAGAAGTACGTTTTCCTGTGGT‐3′; *DNAJC10* forward, 5′‐ GCAGGTTTGACTGTTCCTCTG‐3′ and reverse, 5′‐GGTCCAAGCGTGGTAACATGA‐3′; and *GAPDH* forward, 5′‐TGTGGGCATCAATGGATTTGG‐3′ and reverse, 5′‐ACACCATGTATTCCGGGTCAAT‐3′.

### 2.6 3‐(4,5‐dimethylthiazol‐2‐yl)‐2,5‐diphenyltetrazolium bromide (MTT) assay

LC cells transfected with shRNAs or plasmids were cultured on 6‐well plates at 2000 cells/well for 24, 48, 72, 96 and 120 h. Subsequently, 5 mg/mL MTT solution (20 μL; Sigma) was added in each well for a further 4 h. Then, 100 μL DMSO was added to each well and the OD values at 490 nm were detected using a microplate reader.

### 2.7 Cell colony formation assay

LC cells transfected with shRNAs or plasmids were seeded into 6‐well plates (1000 cells/well) and cultured for 8–14 days, then fixed with 4% paraformaldehyde at 25°C for 30–60 min, followed by addition of 500 μL Giemsa solution to stain cells for 10–20 min at 25°C. Cells were then rinsed several times with double distilled water, and photographed using a camera (Cannon).

### 2.8 Cell cycle analysis

A Cell Cycle Analysis Kit (Sigma) was used to analyse the cell cycle. After harvest, transfected cells were washed using cold PBS, fixed in ice‐cold 95% ethanol at 4°C for 12 h, then incubated in propidium iodide in the dark for 30 min and analysed using a flow cytometer (Agilent Biosciences).

### 2.9 Caspase 3/7 activity

A Homogeneous Caspase‐3/7 Assay (G7790, Promega) was used to assess caspase‐3/7 activity. After cell viability detection via MTT assay, cells were incubated at 25°C for 1 h and fluorescence of each well measured using a FL600 fluorescence plate reader (Bio‐Tek, USA). Caspase‐3/7 activity was calculated as the fluorescence of treated sample/mock control × 100.

### 2.10 Wound‐healing assay

Cell migration was evaluated using a wound‐healing assay. Transfected cells were scratched using 200 μL aseptic pipette to generate a cell‐free area, then washed with PBS, followed by addition of fresh culture medium. Cells were then photographed immediately (0 h) and 24 h later, and the distance between the cells measured to assess cell migration ability.

### 2.11 Transwell assay

Cell migration and invasion were assessed by transwell assay. Briefly, upper chambers of transwell plates were either filled or not with hydrated Matrigel (BD Biosciences) and transfected cells (approximately 5000) in serum‐free 1640 medium, and 500 μL RPMI 1640 medium supplemented with 20% FBS added to the lower chamber. Following incubation at 37°C for 2 days, invasive or migrated cells were fixed, stained with 0.1% crystal violet, and counted under a microscope.

### 2.12 Animal experiments

The Ethics Committee of Peking Union Medical College Hospital approved animal experiments. Female BALB‐c nude mice (24‐week‐old) were purchased from Beijing Vital River Laboratory Animal Technology Co., Ltd. (Beijing, China) and A549 cells transfected with shMCM8 or shCtrl subcutaneously injected into mice to construct xenograft models. Tumour volume was determined each week. Tumours were collected, weighed, and photographed 35 days later, after mice were sacrificed by cervical dislocation. For migration assay in vivo, A549 cells with MCM8 stable knockdown or control cells were injected in to the tail veins of the nude mice (2 × 10^6^ cells per mice). After 42 days of injection, then mice were intraperitoneally injected with D‐luciferin potassium salt (150 mg/kg, BioScience, Shanghai, China) and an in vivo imaging system (IVIS Spectrum, Perkin Elmer) was used to survey fluorescence in mouse bodies.

### 2.13 Gene expression array

TRIzol was used for extraction of total RNA from transfected cells. A Nanodrop 2000 (Thermo) was used to determine RNA concentration and A260/A280 values. A PrimeView Human Gene Expression Array was used to detect gene expression profiles and results analysed using an Affymetrix Scanner 3000 (Affymetrix, USA), with Welch *t*‐test and the Benjamini‐Hochberg false discovery rate (FDR) applied for assessment of raw data. The threshold for statistical significance was set as |fold change| ≥ 1.5 and FDR <0.05.

### 2.14 Mass spectrometry

Total proteins from shMCM8‐ and shCtrl‐transfected cells were lysed, quantified, and separated by 10% SDS‐PAGE. Next, gels were thoroughly rinsed in tissue culture grade water (TCW), then soaked in GelCode Blue Safe Protein Stain (20 mL; Thermo Fisher) for 15 min. Stain was removed by rinsing gels with 20 mL TCW; gels were rinsed with fresh TCW every 10 min for 90 min, then analysed with the Coomassie blue setting on a Bio‐Rad imaging system.

Next, gels were cut into several slices and trypsin (Sigma) used to digest protein bands in‐gel at 37°C overnight. After cleavage and lyophilization, peptides were extracted and dried, then solubilized in water including 2% acetonitrile and 0.5% acetic acid for mass spectrometry analysis by Genechem Co., Ltd. (Shanghai, China).

### 2.15 Co‐immunoprecipitation (Co‐IP) assay

A total of 1.0 mg of proteins were incubated with diluted antibodies at 4°C overnight, followed by addition of 20 μL protein A/G agarose beads (Santa Cruz, CA, USA) and incubation at 4°C for 2 h. IP binding solution was used to clean the precipitates after protein centrifugation. IP lysate buffer (Pierce, Thermo Fisher Scientific, Cat. No. 87787) in 5 × loading buffer was used to denature the proteins at 100°C in boiling water for 5 min. Western blot assays were performed using the obtained reactants and corresponding antibodies.

### 2.16 Statistical analysis

Data are presented as the mean ± standard deviation and were analysed using SPSS version 16.0. Differences among groups were determined using the Student's *t*‐test. Spearman correlation was employed to analyse expression trends at various stages. *p* < 0.05 was considered significant.

## 3 RESULTS

### 3.1 MCM8 is upregulated in cancer tissue and cells

**FIGURE 1:**
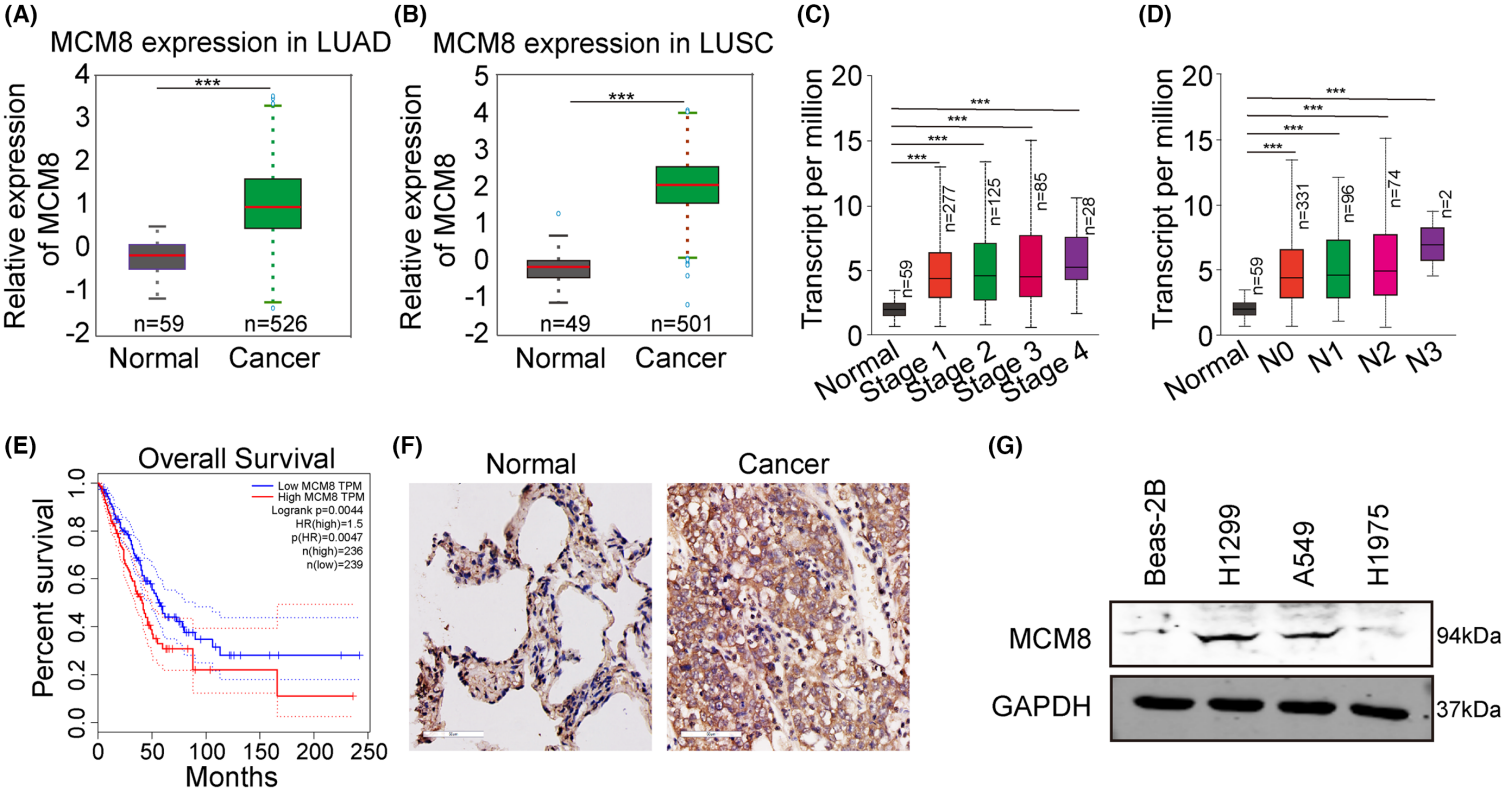
MCM8 is highly expressed in cancer tissue and cells. (A, B) MCM8 expression in LUAD and LUSC samples from TCGA database. (C, D) Relationships of *MCM8* mRNA levels with tumour stage and regional lymph node metastasis. (E) Relationship between *MCM8* expression and overall survival of patients with LC. (F) MCM8 expression in tumour samples from patients with LC detected by immunohistochemistry staining. (G) MCM8 expression in three LC cell lines and normal lung cells determined by western blot. ***p < 0.001.

To investigate the role of MCM8 in LC, *MCM8* expression in LC and normal tissue samples from TCGA database was first analysed, and we found that it was significantly upregulated in both LUAD and LUSC samples, relative to normal lung tissue controls (Figure 1A,B). *MCM8* expression level was associated with tumour stage and regional lymph node metastasis (Figure 1C,D). Further, Kaplan–Meier analysis demonstrated that high *MCM8* expression was an indicator of shorter OS of patients with LC (Figure 1E). Furthermore, IHC staining showed higher MCM8 signal intensity in LC relative to paracancerous tissue (Figure 1F; Table S1). Western blot analysis of MCM8 in three LC cell lines showed that MCM8 was frequently upregulated compared to levels in normal lung cells (Beas‐2B) (Figure 1G). These results indicate that MCM8 is expressed at high levels in primary LC tumours and LC cell lines.

### 3.2 Silencing of MCM8 suppresses cell proliferation and migration

**FIGURE 2:**
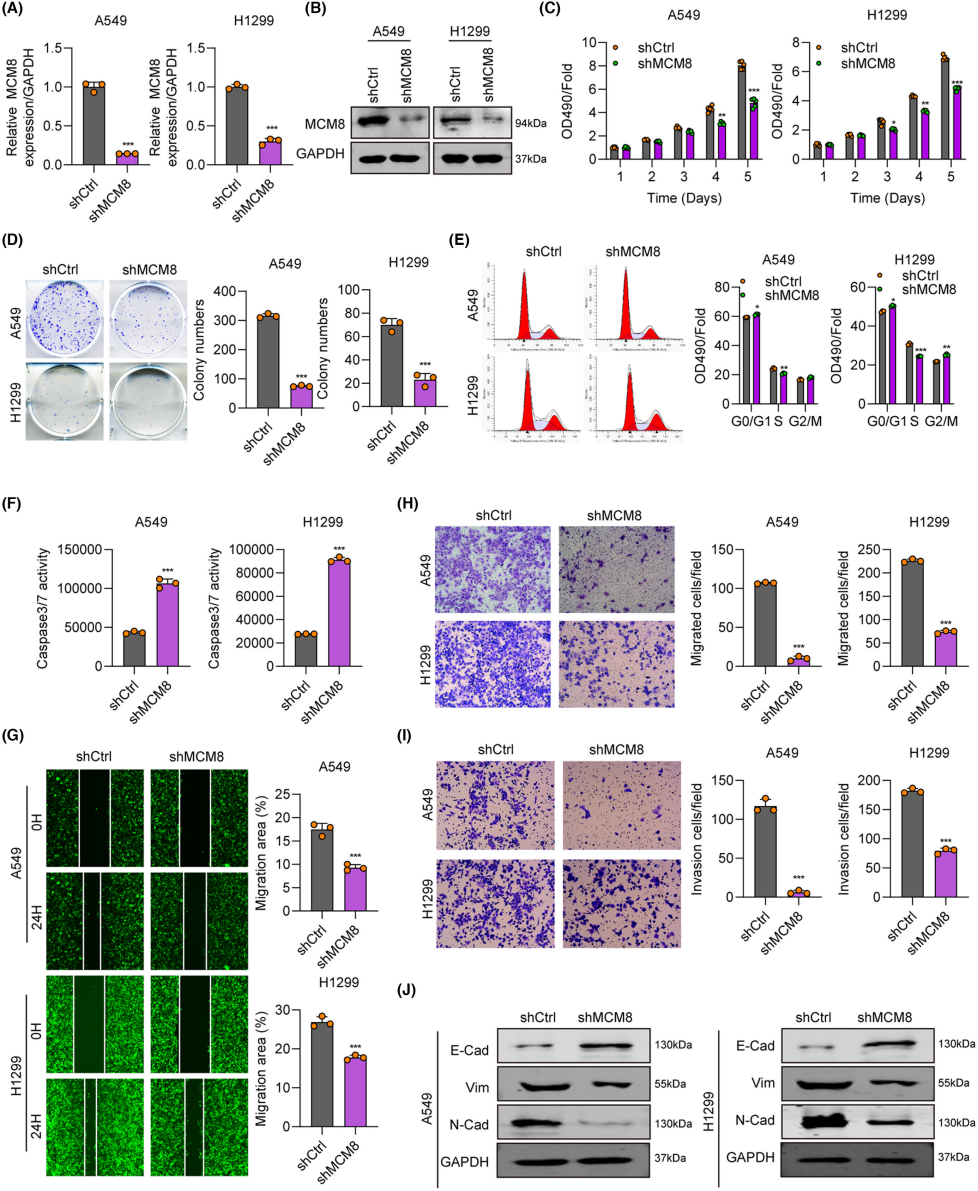
Silencing of MCM8 suppresses cell proliferation and migration. (A, B) MCM8 knockdown efficiency in LC cells evaluated by RT‐qPCR and western blot. (C) Cell proliferation rate after MCM8 knockdown evaluated by MTT assay. (D) LC cell colony numbers after MCM8 knockdown analysed by cell colony formation assay. (E) Cell cycle analysis by flow cytometry after MCM8 knockdown. (F) Apoptosis after MCM8 knockdown, determined by assessing Caspase 3/7 activity. (G) Cell migration in shCtrl‐ and shMCM8‐treated cells, determined by wound‐healing assay. (H, I) Cell migration and invasion in shCtrl‐ and shMCM8‐treated cells, determined by transwell assay. (J) N‐cadherin, E‐cadherin, and Vimentin expression after MCM8 knockdown, detected by western blot. *p < 0.05, **p < 0.01, ***p < 0.001.

To explore the role of MCM8 in LC, we transfected A549 and H1299 cells with shMCM8 or shCtrl. Compared with shCtrl cells, MCM8 mRNA and protein levels were all markedly reduced in A549 and H1299 cells transfected with shMCM8 (Figure 2A,B). MTT and cell colony formation assays showed that shMCM8 treatment decreased cell replication relative to cells treated with shCtrl (Figure 2C,D). Further, flow cytometry analysis revealed that MCM8 depletion in two LC cell lines led to a reduction of S phase cells, while G0/G1 phase cells were increased (Figure 2E). Additionally, the percentage of Caspase 3/7 (indicating apoptosis) was higher in the shMCM8 group compared with the shCtrl group (Figure 2F). Wound‐healing assays indicated attenuated cell migration in the shMCM8 group (Figure 2G). We also performed transwell assays to confirm the effects of MCM8 on cell migration, and found that cell invasion was blocked by MCM8 knockdown (Figure 2H,I). Subsequently, the EMT‐related proteins, N‐cadherin and Vimentin, were found to be downregulated in A549 and H1299 cells after MCM knockdown, while E‐cadherin was upregulated (Figure 2J). Overall, our results indicate that silencing of MCM8 attenuated LC cell proliferation and migration.

### 3.3 Overexpression of MCM8 promotes cell proliferation and migration

**FIGURE 3:**
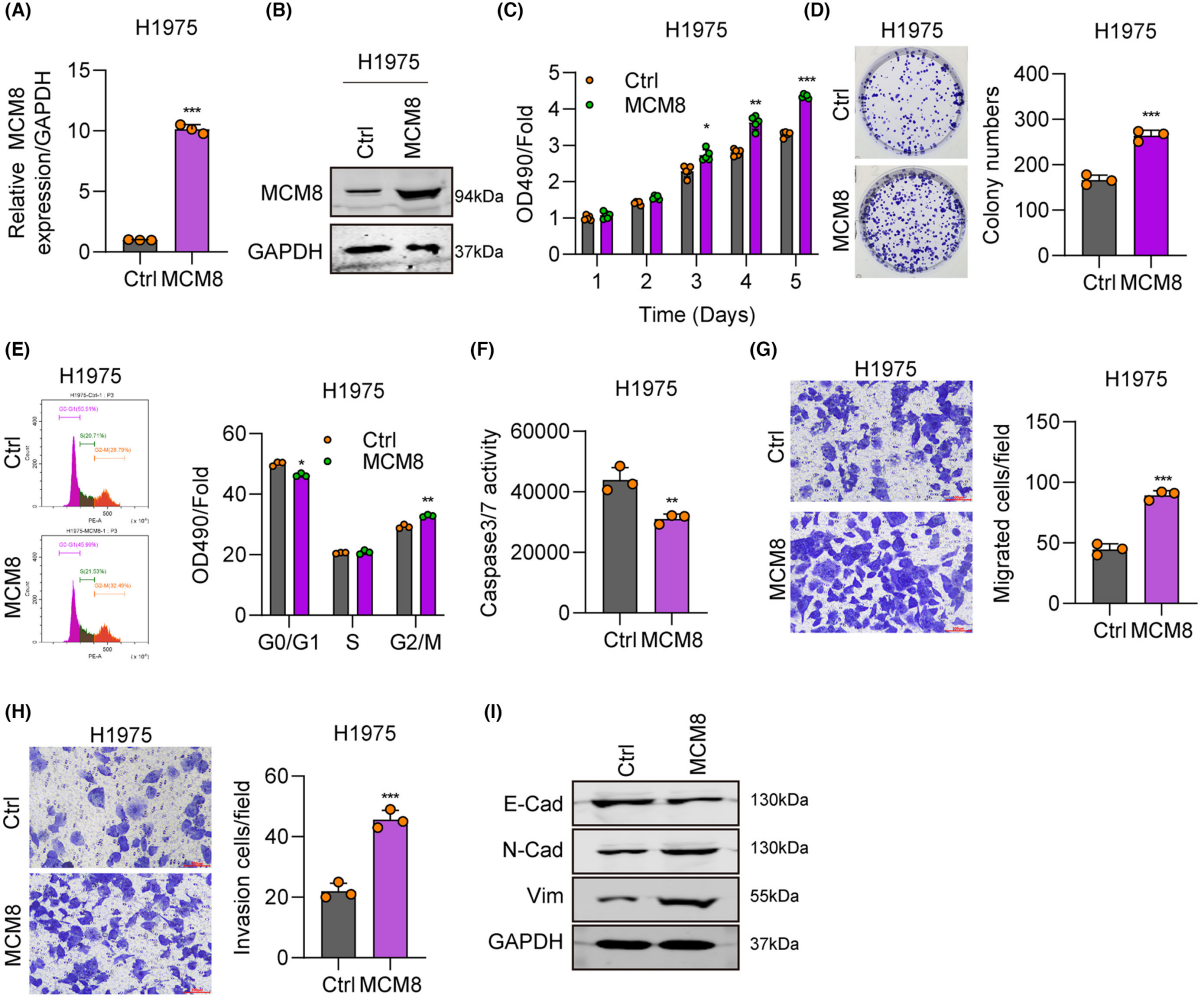
MCM8 overexpression promotes cancer cell proliferation and migration. (A, B) MCM8 overexpression efficiency in LC cells, determined by RT‐qPCR and western blot. (C) Cell proliferation rates following MCM8 overexpression, evaluated by MTT assay. (D) Cell colony numbers following MCM8 overexpression, analysed by cell colony formation assay. (E) Cell cycle changes following MCM8 overexpression, determined by flow cytometry. (F) Apoptosis following MCM8 overexpression, based on Caspase 3/7 activity. (G, H) Cell migration and invasion ability following MCM8 overexpression, detected by transwell assay. (I) N‐cadherin, E‐cadherin, and Vimentin expression levels on MCM8 overexpression, detected by western blot. *p < 0.05, **p < 0.01, ***p < 0.001.

After transfection with MCM8‐overexpression plasmids, MCM8 mRNA and protein levels were markedly upregulated, as demonstrated by RT‐qPCR and western blot assays (Figure 3A,B). MTT and cell colony formation assays demonstrated that MCM8 overexpression significantly increased cell viability and colony numbers in H975 cell lines (Figure 3C,D). As shown in Figure 3E, MCM8 overexpression led to an increase in the cell population in G2/M phase and a corresponding decrease in G0/G1 phase cells. Further, compared with the control group, Caspase 3/7 activity was greatly decreased in H1975 cell lines overexpressing MCM8 (Figure 3F). Transwell assays demonstrated considerably greater invasion and migration of H1975 cells after transfection of MCM8 overexpression plasmids (Figure 3G,H). N‐cadherin and Vimentin expression were upregulated, and that of E‐cadherin was downregulated following MCM8 overexpression in H1975 cells (Figure 3I). Overall, MCM8 upregulation was observed to promote LC cell proliferation, migration, and invasion.

### 3.4 MCM8 knockdown suppresses LC tumorigenesis and migration in vivo

**FIGURE 4:**
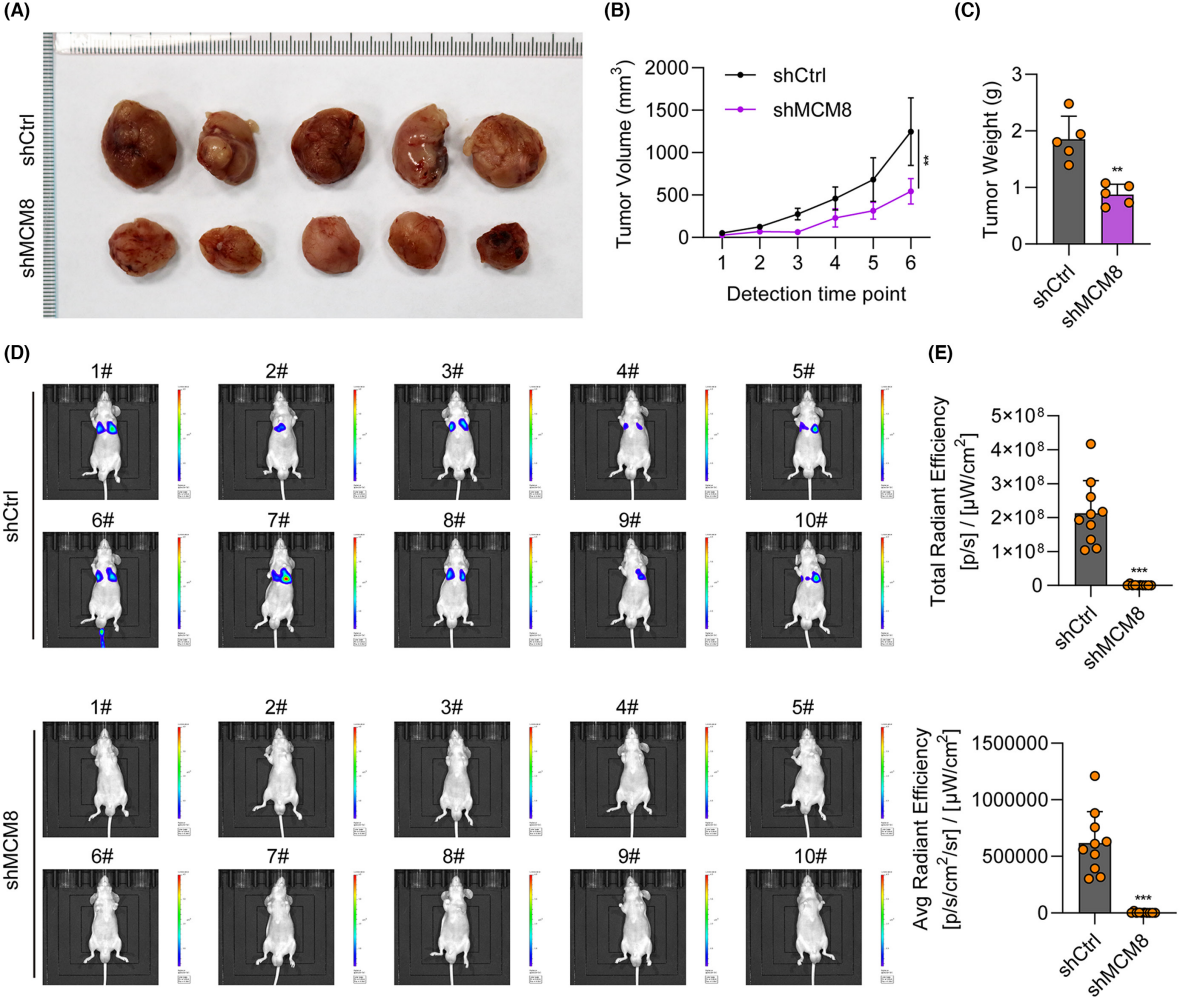
MCM8 knockdown suppresses growth and migration in vivo. (A) Construction of a nude mouse xenograft model using cells transfected with shMCM8 and shCtrl. Tumours were photographed after their removal at 35 days. (B, C) Tumour growth curves and comparison of tumour weights between the shCtrl and shMCM8 groups. (D, E) Comparison of fluorescence intensity in mice in the shMCM8 and shCtrl groups. **p < 0.01, ***p < 0.001.

In vivo nude mice models were used to further explore the role of MCM8 in LC tumorigenesis. We subcutaneously injected MCM8‐depleted A549 cells into mice. Tumour size in mice injected with cells transfected with shCtrl and shMCM8 is shown in Figure 4A; tumours were smaller in the shMCM8 group than those in the shCtrl group. Further, tumour volume and weight were significantly reduced in animals with shMCM8‐treated tumours (Figure 4B,C). To confirm the role of MCM8 in cell migration, the A549 cells transfected with shMCM8 or shCtrl were injected into the tail veins of the nude mice. After 42 days later, the migration of A549 cells transfected with shMCM8 or shCtrl were measured using a in vivo imaging system. As expected, in vivo imaging revealed decreased fluorescence in the shMCM8 group, indicating reduced tumour growth (Figure 4D,E). These results indicate that MCM8 knockdown suppressed LC growth and migration in vivo.

### 3.5 MCM8 upregulates and interacts with DNAJC10

**FIGURE 5:**
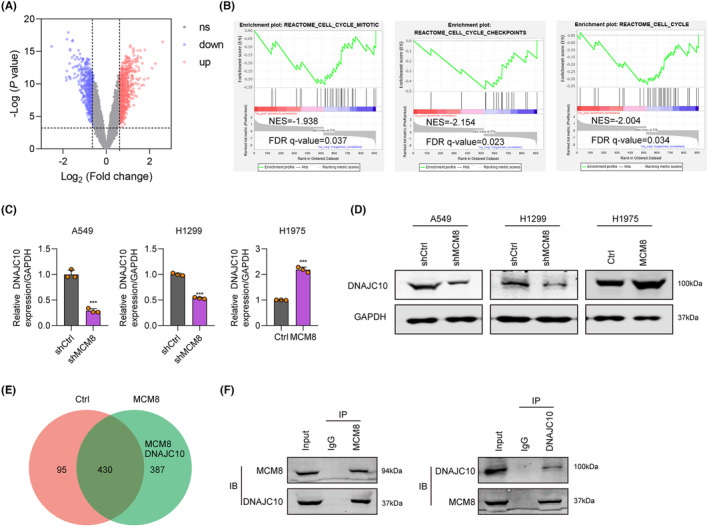
MCM8 upregulates and interacts with DNAJC10. (A) Volcano plot of DEGs between cells treated with shCtrl or shMCM8. (B) GSEA snapshots of KEGG pathway enrichment analysis. (C, D) DNAJC10 expression determined by (C) qRT‐PCR and (D) western blot in A549 and H1299 cells treated with shMCM8 and H1975 cells overexpressing MCM8. (E) DEG interaction network diagram generated using IPA. (F) Interaction between MCM8 and DNAJC10 verified by Co‐IP assay. ***p < 0.001.

To identify the mechanism downstream of MCM8 in LC, Gene Array analysis was implemented to search for differentially expressed genes (DEGs) in A549 cells treated with shMCM8 or shCtrl. As shown in Figure 5A, there were 519 upregulated and 393 downregulated DEGs (fold change ≥1.5) after MCM8 knockdown. GSEA analysis showed that cell cycle, cell cycle mitotic, and cell cycle checkpoint were the most significantly enriched pathways (*p* < 0.05, Figure 5B). Furthermore, IPA analysis revealed a DEG interaction network, and DNAJC10 was found to interact with MCM8 (Figure 5E). DNAJC10 mRNA (Figure 5C) and protein (Figure 5D) levels were significantly decreased in shMCM8‐treated A549 and H1299 cells, while increased in MCM8 overexpressed H1975 cells, as determined by qPCR and western blot. Finally, co‐IP assay demonstrated that DNAJC10 levels were significantly higher in the MCM8‐Flag group, while those of MCM8 were elevated in the DNAJC10‐Flag group (Figure 5F), indicating that there was an interaction between DNAJC10 and MCM8. These data suggest that DNAJC10 is a downstream target of MCM8 in LC.

### 3.6 MCM8 promotes LC growth and migration through upregulating DNAJC10

**FIGURE 6:**
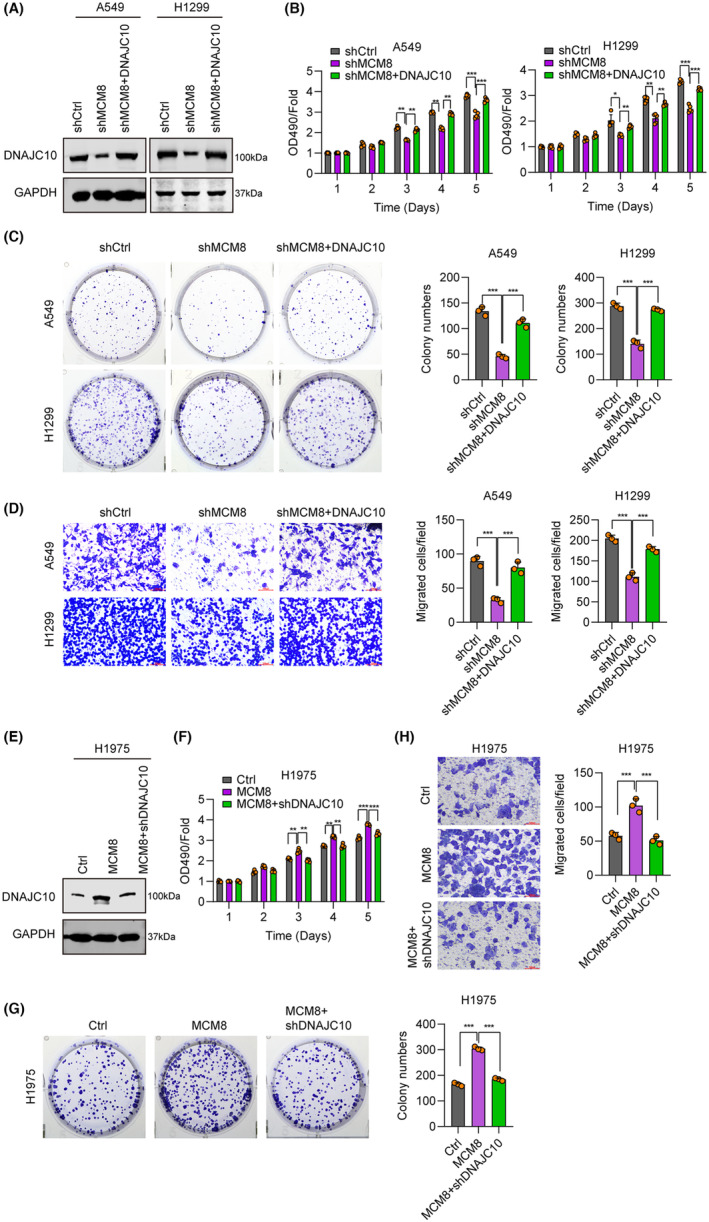
MCM8 promotes lung cancer growth and migration by upregulating DNAJC10. (A) DNAJC10 expression in A549 and H1299 cells transfected with shCtrl, shMCM8, or shMCM8+DNAJC10, detected by western blot. (B) MTT assay of cell proliferation in the three groups. (C) Colony formation assay to determine cell colony numbers in the three groups. (D) Transwell assays to determine cell migration ability in the three groups. (E) DNAJC10 expression in H1975 cells transfected with Ctrl, MCM8, or MCM8+shDNAJC10, determined by western blot. (F) MTT assay of cell proliferation in the MCM8 and MCM8 + shDNAJC10 groups. (G) Colony formation assay in the MCM8 and MCM8+shDNAJC10 groups. (H) The results of transwell assays in the MCM8 and MCM8+shDNAJC10 groups. *p < 0.05, **p < 0.01, ***p < 0.001.

Recovery assays using shMCM8 and DNAJC10 were used to test the synergistic function of DNAJC10 and MCM8 in LC progression. DNAJC10 protein levels were decreased in the shMCM8 group, while they were recovered to normal levels in the shMCM8+DNAJC10 group (Figure 6A). Compared with the shMCM8 group, cell proliferation and migration were also recovered to normal levels in the shMCM8+DNAJC10 group (Figure 6B–D). Furthermore, H1975 cells overexpressing MCM8 with or without shDNAJC10 were generated, and we found that DNAJC10 protein levels were substantially increased in the MCM8 group, whereas they were reduced to control levels in the MCM8+shDNAJC10 group (Figure 6E). Overexpression of MCM8 alone increased cell viability (Figure 6F), cell migration (Figure 6H), and colony formation (Figure 6G), while DNAJC10 knockdown partially reverse these effects in LC cells, clearly implicating DNAJC10 as a potential target of MCM8 in LC.

### 3.7 MCM8 levels are positively correlated with those of DNAJC10 in patients with LC

**FIGURE 7:**
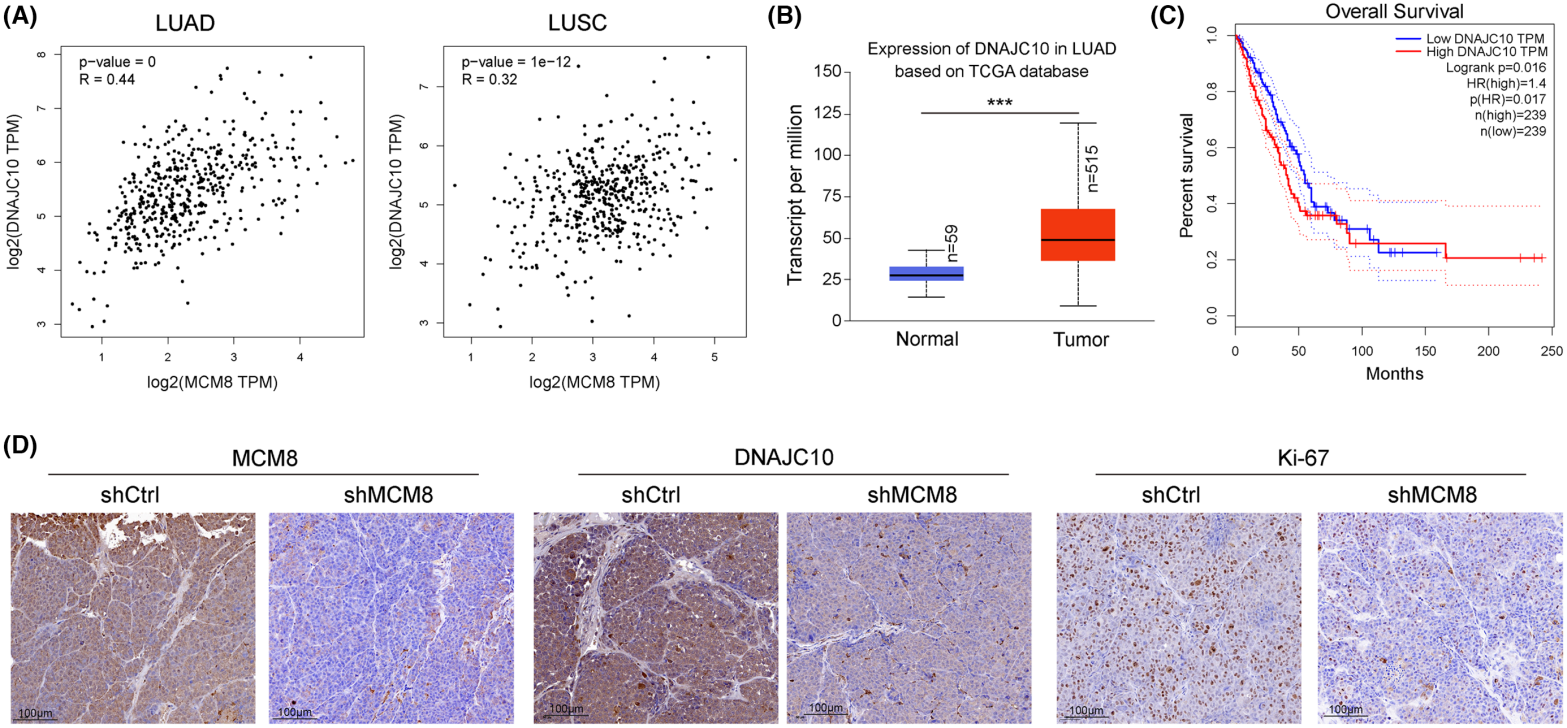
MCM8 levels are positively correlated with those of DNAJC10 in LC samples. (A) Correlation of *MCM8* and *DNAJC10* mRNA levels in LUAD and LUSC samples from TCGA database. (B) *DNAJC10* expression in LUAD samples from TCGA database. (C) Higher *DNAJC10* expression was associated with poor overall survival of patients with LC. ***p < 0.001. (D) MCM8, DNAJC10 and Ki67expression in tumour samples from patients with LC detected by immunohistochemistry staining.

Correlation between DNAJC10 and MCM8 expression was investigated using LUAD and LUSC data from TCGA database and remarkable positive correlations were observed (*R* = 0.44 in LUAD and *R* = 0.32 in LUSC; Figure 7A). DNAJC10 was upregulated in LUAD samples relative to normal controls (Figure 7B). Furthermore, high DNAJC10 expression was associated with shorter OS in patients with LC, according to Kaplan–Meier analysis (*p* = 0.017, Figure 7C). Furthermore, our IHC staining analysis showed that the expression of MCM8, DNAJC10 and Ki67 were all decreased in MCM8 knock down xenograft model‐derived tumours compared with control (Figure 7D). These findings suggest a positive regulatory relationship between MCM8 and DNAJC10.

## 4 DISCUSSION

The MCM protein family was first identified in the budding yeast, *Saccharomyces cerevisiae*,^23^ as molecules involved in DNA replication.^24^ The human MCM8 protein contains a central MCM domain, and is expressed in many cell lines and human tissues.^25^ MCM2, MCM4, and MCM7 regulate LC cell proliferation, and may act as prognostic markers in patients with LC.^26^, ^27^, ^28^ Li et al.^21^ found that *MCM2*, *MCM4*, and *MCM10* expression levels were dramatically elevated in patients with LUAD, based on comprehensive mRNA profiling of MCM family members; high mRNA expression of *MCM2–5*, *MCM8*, and *MCM10* were related to inferior OS and disease‐free survival. Liu et al.^20^ assess the potential roles of *MCM1*–*10* as diagnostic biomarkers in patients with LUAD, and found that *MCM4*, *MCM5*, and *MCM8* may be promising prognostic indicators of LUAD using a genome‐wide RNA sequencing dataset and bioinformatics analyses.

Here, we found that MCM8 levels are higher in LC tissues than those in control samples from both TCGA database and clinical patients. Besides, higher MCM8 levels were associated with advanced tumour stage and lymph node metastasis, as well as poor OS, consistent with the findings of Li et al.^21^ and Liu et al.^20^ Functional analyses showed that MCM8 is highly expressed in three LC cell lines compare to normal lung cells (Beas‐2B). MCM8 knockdown suppressed cell proliferation, migration, and invasion, and led to LC cell cycle arrest, while MCM8 overexpression had the opposite effects. Animal experiments also confirmed the oncogenic role of MCM8 in nude mice with LC xenografts. To explore the functional mechanism involved in the effects of MCM8 on LC progression, we conducted gene array analysis to scan for DEGs between A549 cells with MCM8 knocked down and control A549 cells; 519 upregulated and 393 downregulated DEGs were identified. GSEA analysis showed enrichment for cell cycle functions in LC progression, with the heat shock protein, DNAJC10, ranking first in MCM8‐depleted cells. Altered DNAJC10 mRNA and protein levels were detected in cells with MCM8 overexpressed or knocked down. Furthermore, CO‐IP assay confirmed interaction between the MCM8 and DNAJC10 proteins. As far as we know, this is the first research to investigate the effects of MCM8/DNAJC10 axis in LC progression.

DnaJ heat shock protein family (Hsp40) member C10 (DNAJC10), also known as ERDJ5 or PDIA19, is the largest member of the protein disulfide isomerase family, with a single N‐terminal J domain and six thioredoxin‐like domains.^29^ DNAJC10 encodes an endoplasmic reticulum (ER) localized protein that forms an ER‐associated degradation (ERAD) complex, involved in recognizing and degrading misfolded proteins.^30^ Recently, DNAJC10 was reported to participate in glioma,^31^ breast cancer,^32^ and neuroblastoma.^33^ Further, the significance of DNAJC10 expression varies among different cancers; for example, high DNAJC10 expression is associated with poor prognosis in patients with glioma,^31^ while *DNAJC10* mRNA downregulation predicts weak OS and relapse‐free survival in patients with breast cancer.^32^ DNAJC10 is also reported to be involved in LUAD cell proliferation and invasiveness.^34^

We also conducted recovery assays, using DNAJC10 plasmids or shRNAs to investigate the mechanism underlying the effects of MCM8 on LC development. Western blots verified DNAJC10 overexpression or knockdown efficiency. MTT, cell colony formation, and transwell assays were applied to measure cell proliferation and migration capacity, and the results revealed that MCM8 knockdown suppressed cell development, while addition of DNAJC10 overexpression plasmids recovered cell proliferation and migration of MCM8‐deficient cells. Hence, DNAJC10 knockdown partially reversed the effects of MCM8 overexpression on LC cells. Subsequently, we detected a remarkable positive correlation between DNAJC10 and MCM8 expression using TCGA database and clinical data. We also observed a relationship between high DNAJC10 expression and poor OS in patients with LC, consistent with a report of the effect of DNAJC10 in glioma.^31^ The limitation of the study is whether or how the effect of DNAJC10 in LC progression in MCM8 knock down mice model.

## 5 CONCLUSIONS

In summary, our findings showed that MCM8 appears to have an oncogenic role in LC progression, and the MCM8/DNAJC10 axis is a potential target in LC therapy. Furthermore, high expression of MCM8 and DNAJC10 are both indicative of poor OS in patients with LC, suggesting that they could serve as prognostic markers in this study.

## AUTHOR CONTRIBUTIONS

**Lei Cao:** Investigation (equal); methodology (equal); validation (equal); writing – original draft (equal). **Hongsheng Liu:** Investigation (equal); methodology (equal); validation (equal); writing – review and editing (equal). **Zhijun Han:** Investigation (equal); methodology (equal); validation (equal); writing – review and editing (equal). **Cheng Huang:** Investigation (equal); methodology (equal); validation (equal); writing – review and editing (equal). **Chao Guo:** Formal analysis (equal); investigation (equal); resources (equal); writing – review and editing (equal). **Luo Zhao:** Formal analysis (equal); investigation (equal); resources (equal); writing – review and editing (equal). **Chao Gao:** Formal analysis (equal); investigation (equal); resources (equal); writing – review and editing (equal). **Yuan Xu:** Data curation (equal); formal analysis (equal); software (equal); writing – review and editing (equal). **Guige Wang:** Data curation (equal); formal analysis (equal); software (equal); writing – review and editing (equal). **Zhe Feng:** Formal analysis (equal); investigation (equal); resources (equal); writing – review and editing (equal). **Shanqing Li:** Conceptualization (lead); funding acquisition (lead); investigation (lead); writing – review and editing (lead).

## FUNDING INFORMATION

This study was supported by National High Level Hospital Clinical Research Funding, No. 2022‐PUMCH‐B‐011.

## CONFLICT OF INTEREST STATEMENT

The authors declare that there are no conflicts of interests.

### ETHICS STATEMENT

This study was approved by the Ethics Committee of Peking Union Medical College Hospital, Peking Union Medical College and Chinese Academy of Medical Sciences, for studying involving humans (S‐K1083). The animal study was approved by the Ethics Committee of Peking Union Medical College Hospital, Peking Union Medical College and Chinese Academy of Medical Sciences (S‐K1083).

### CONSENT

Informed consent was obtained from all subjects involved in the study.

## Supporting information


Table S1.

## Data Availability

DATA AVIALABILITY STATEMENT Data can be available upon request.

## References

[1] Thai AA , Solomon BJ , Sequist LV , Gainor JF , Heist RS . Lung cancer. Lancet. 2021;398:535‐554. 34273294 10.1016/S0140-6736(21)00312-3

[2] Luo Q , Li X , Meng Z , et al. Identification of hypoxia‐related gene signatures based on multi‐omics analysis in lung adenocarcinoma. J Cell Mol Med. 2024;28(2):e18032. 38013642 10.1111/jcmm.18032 PMC10826438

[3] Yu Z , Tang H , Chen S , et al. Exosomal LOC85009 inhibits docetaxel resistance in lung adenocarcinoma through regulating ATG5‐induced autophagy. Drug Resist Updat. 2023;67:100915. 36641841 10.1016/j.drup.2022.100915

[4] Nooreldeen R , Bach H . Current and future development in lung cancer diagnosis. Int J Mol Sci. 2021;22(16):8661. 34445366 10.3390/ijms22168661 PMC8395394

[5] Miller KD , Siegel RL , Lin CC , et al. Cancer treatment and survivorship statistics, 2016. CA Cancer J Clin. 2016;66:271‐289. 27253694 10.3322/caac.21349

[6] Du W , Yin F , Zhong Y , et al. CircUCP2 promotes the tumor progression of non‐small cell lung cancer through the miR‐149/UCP2 pathway. Oncol Res. 2023;31:929‐936. 37744277 10.32604/or.2023.030611 PMC10513941

[7] Ruiz‐Cordero R , Devine WP . Targeted therapy and checkpoint immunotherapy in lung cancer. Surg Pathol Clin. 2020;13:17‐33. 32005431 10.1016/j.path.2019.11.002

[8] Xie G , Li Y , Jiang Y , Ye X , Tang J , Chen J . Silencing HIPPI suppresses tumor progression in non‐small‐cell lung cancer by inhibiting DNA replication. Onco Targets Ther. 2021;14:3467‐3480. 34079292 10.2147/OTT.S305388 PMC8166357

[9] Kearsey SE , Labib K . MCM proteins: evolution, properties, and role in DNA replication. Biochim Biophys Acta. 1998;1398:113‐136. 9689912 10.1016/s0167-4781(98)00033-5

[10] Maiorano D , Lutzmann M , Mechali M . MCM proteins and DNA replication. Curr Opin Cell Biol. 2006;18:130‐136. 16495042 10.1016/j.ceb.2006.02.006

[11] Guilbaud G , Sale JE . Unwinding to recombine. Mol Cell. 2012;47:493‐494. 22920289 10.1016/j.molcel.2012.08.006

[12] Freeman A , Morris LS , Mills AD , et al. Minichromosome maintenance proteins as biological markers of dysplasia and malignancy. Clin Cancer Res. 1999;5:2121‐2132. 10473096

[13] Zhong X , Chen X , Guan X , et al. Overexpression of G9a and MCM7 in oesophageal squamous cell carcinoma is associated with poor prognosis. Histopathology. 2015;66:192‐200. 24805087 10.1111/his.12456

[14] Liu Z , Li J , Chen J , et al. MCM family in HCC: MCM6 indicates adverse tumor features and poor outcomes and promotes S/G2 cell cycle progression. BMC Cancer. 2018;18:200. 29463213 10.1186/s12885-018-4056-8 PMC5819696

[15] Das M , Prasad SB , Yadav SS , et al. Over expression of minichromosome maintenance genes is clinically correlated to cervical carcinogenesis. PLoS One. 2013;8:e69607. 23874974 10.1371/journal.pone.0069607 PMC3714251

[16] Giaginis C , Giagini A , Tsourouflis G , et al. MCM‐2 and MCM‐5 expression in gastric adenocarcinoma: clinical significance and comparison with Ki‐67 proliferative marker. Dig Dis Sci. 2011;56:777‐785. 20694513 10.1007/s10620-010-1348-5

[17] Hao J , Deng H , Yang Y , et al. Downregulation of MCM8 expression restrains the malignant progression of cholangiocarcinoma. Oncol Rep. 2021;46(5):235. 34523691 10.3892/or.2021.8186 PMC8453687

[18] Wang X , Zhang L , Song Y , et al. MCM8 is regulated by EGFR signaling and promotes the growth of glioma stem cells through its interaction with DNA‐replication‐initiating factors. Oncogene. 2021;40:4615‐4624. 34131285 10.1038/s41388-021-01888-1

[19] Ren Z , Li J , Zhao S , Qiao Q , Li R . Knockdown of MCM8 functions as a strategy to inhibit the development and progression of osteosarcoma through regulating CTGF. Cell Death Dis. 2021;12:376. 33828075 10.1038/s41419-021-03621-y PMC8027380

[20] Liu K , Kang M , Liao X , Wang R . Genome‐wide investigation of the clinical significance and prospective molecular mechanism of minichromosome maintenance protein family genes in patients with lung adenocarcinoma. PLoS One. 2019;14:e0219467. 31323040 10.1371/journal.pone.0219467 PMC6641114

[21] Li S , Jiang Z , Li Y , Xu Y . Prognostic significance of minichromosome maintenance mRNA expression in human lung adenocarcinoma. Oncol Rep. 2019;42:2279‐2292. 31545501 10.3892/or.2019.7330 PMC6826304

[22] Livak KJ , Schmittgen TD . Analysis of relative gene expression data using real‐time quantitative PCR and the 2(‐Delta Delta C(T)) method. Methods. 2001;25:402‐408. 11846609 10.1006/meth.2001.1262

[23] Zhang T , Chen G , Liu C , et al. A phase I study comparing the pharmacokinetics, safety, and immunogenicity of proposed biosimilar GB242 and reference infliximab in healthy subjects. BioDrugs. 2019;33:93‐100. 30511316 10.1007/s40259-018-0326-x

[24] Zhai Y , Li N , Jiang H , Huang X , Gao N , Tye BK . Unique roles of the non‐identical MCM subunits in DNA replication licensing. Mol Cell. 2017;67:168‐179. 28732205 10.1016/j.molcel.2017.06.016

[25] Gozuacik D , Chami M , Lagorce D , et al. Identification and functional characterization of a new member of the human mcm protein family: hMcm8. Nucleic Acids Res. 2003;31:570‐579. 12527764 10.1093/nar/gkg136 PMC140502

[26] Werynska B , Pula B , Muszczynska‐Bernhard B , et al. Correlation between expression of metallothionein and expression of Ki‐67 and MCM‐2 proliferation markers in non‐small cell lung cancer. Anticancer Res. 2011;31:2833‐2839. 21868526

[27] Wu W , Wang X , Shan C , Li Y , Li F . Minichromosome maintenance protein 2 correlates with the malignant status and regulates proliferation and cell cycle in lung squamous cell carcinoma. Onco Targets Ther. 2018;11:5025‐5034. 30174440 10.2147/OTT.S169002 PMC6109654

[28] Fujioka S , Shomori K , Nishihara K , et al. Expression of minichromosome maintenance 7 (MCM7) in small lung adenocarcinomas (pT1): prognostic implication. Lung Cancer. 2009;65:223‐229. 19144445 10.1016/j.lungcan.2008.11.007

[29] Maegawa KI , Watanabe S , Noi K , et al. The highly dynamic nature of ERdj5 is key to efficient elimination of aberrant protein oligomers through ER‐associated degradation. Structure. 2017;25(846–57):e4. 10.1016/j.str.2017.04.001 28479060

[30] Ushioda R , Hoseki J , Araki K , Jansen G , Thomas DY , Nagata K . ERdj5 is required as a disulfide reductase for degradation of misfolded proteins in the ER. Science. 2008;321:569‐572. 18653895 10.1126/science.1159293

[31] Liu F , Tu Z , Liu J , et al. DNAJC10 correlates with tumor immune characteristics and predicts the prognosis of glioma patients. Biosci Rep. 2022;42(1):BSR20212378. 34988580 10.1042/BSR20212378 PMC8766825

[32] Acun T , Senses KM . Downregulation of DNAJC10 (ERDJ5) is associated with poor survival in breast cancer. Breast Cancer. 2020;27:483‐489. 31902119 10.1007/s12282-019-01042-6

[33] Thomas CG , Spyrou G . ERdj5 sensitizes neuroblastoma cells to endoplasmic reticulum stress‐induced apoptosis. J Biol Chem. 2009;284:6282‐6290. 19122239 10.1074/jbc.M806189200

[34] Lou Y , Lu J , Zhang Y , et al. The centromere‐associated protein CENPU promotes cell proliferation, migration, and invasiveness in lung adenocarcinoma. Cancer Lett. 2022;532:215599. 35176420 10.1016/j.canlet.2022.215599

