# Supplementary material for: MCM8 promotes lung cancer progression through upregulating DNAJC10
Source: J Cell Mol Med. 2024 Jun 21;28(12):e18488. doi: 10.1111/jcmm.18488 (PMC11190951; doi:10.1111/jcmm.18488)
Supplement: Supplementary file 1 — Table S1. [file JCMM-28-e18488-s001.doc]

Table 1. The expression of MCM8 in lung cancer and normal tissues

| Type | MCM8 expression | | [χ^2^](https://www.baidu.com/s?rsv_idx=1&tn=99963492_hao_pg&wd=χ2分布公式&fenlei=256&usm=1&ie=utf-8&rsv_pq=98791d880002a41c&oq=卡方&rsv_t=6e2dPw%2FeUZ48NOmczH4QoKN%2BldDc82gpYoSXp2YbPlCPHI5iAoYnHNg6EDVfVUybCW9iqS8E&rsf=11630013&rsv_dl=0_prs_28608_1) | P-value |
| --- | --- | --- | --- | --- |
|  | High | Low |  |  |
| Normal | 11 | 17 | 15.525 | 8.14*10^-5^ |
| Lung cancer | 219 | 75 |  |  |
